# Deep learning and radiomics-driven algorithm for automated identification of May-Thurner syndrome in Iliac CTV imaging

**DOI:** 10.3389/fmed.2025.1526144

**Published:** 2025-04-29

**Authors:** Lufeng Chen, Dong-Lin Li, Hua-Feng Zheng, Cheng-Zhi Qiu

**Affiliations:** ^1^Department of Thoracic and Cardiovascular Surgery, The Second Affiliated Hospital of Fujian Medical University, Quanzhou, Fujian, China; ^2^Department of General Surgery, The Second Affiliated Hospital of Fujian Medical University, Quanzhou, Fujian, China

**Keywords:** May-Thurner syndrome, deep learning, Computed Tomography Venography, iliac vein compression, Convolutional Neural Networks

## Abstract

**Objective:**

This research aimed to create a dataset of Iliac CTV scans for automated May-Thurner syndrome (MTS) detection using deep learning and radiomics. In addition, it sought to establish an automated segmentation model for Iliac Vein CTV scans and construct a radiomic signature for MTS diagnosis.

**Methods:**

We collected a dataset of 490 cases meeting specific inclusion and exclusion criteria, anonymized to comply with HIPAA regulations. Iliac Vein CTV scans were prepared with contrast agent administration, followed by image acquisition and evaluation. A deep learning-based segmentation model, UPerNet, was employed using 10-fold cross-validation. Radiomic features were extracted from the scans and used to construct a diagnostic radiomic signature. Statistical analysis, including Dice values and ROC analysis, was conducted to evaluate segmentation and diagnostic performance.

**Results:**

The dataset consisted of 201 positive cases of MTS and 289 negative cases. The UPerNet segmentation model exhibited remarkable accuracy in identifying MTS regions. A Dice coefficient of 0.925 (95% confidence interval: 0.875–0.961) was observed, indicating the precision and reliability of our segmentation model. Radiomic analysis produced a diagnostic radiomic signature with significant clinical potential. ROC analysis demonstrated promising results, underscoring the efficacy of the developed model in distinguishing MTS cases. The radiomic signature demonstrated strong diagnostic capabilities for MTS. Within the training dataset, it attained a notable area under the curve (AUC) of 0.891, with a 95% confidence interval ranging from 0.825 to 0.956, showcasing its effectiveness. This diagnostic capability extended to the validation dataset, where the AUC remained strong at 0.892 (95% confidence interval: 0.793–0.991). These results highlight the accuracy of our segmentation model and the diagnostic value of our radiomic signature in identifying MTS cases.

**Conclusion:**

This study presents a comprehensive approach to automate MTS detection from Iliac CTV scans, combining deep learning and radiomics. The results suggest the potential clinical utility of the developed model in diagnosing MTS, offering a non-invasive and efficient alternative to traditional methods.

## Introduction

May-Thurner syndrome (MTS), also known as iliac vein compression syndrome, is a condition that frequently goes underdiagnosed. This syndrome is associated with significant morbidity, predominantly due to its correlation with deep vein thrombosis (DVT), especially in the left lower extremity (1). The pathophysiological basis involves the compression of the left common iliac vein by the overriding right common iliac artery, first described in cadaveric studies by May and Thurner (2). This physiological condition is characterized by the compression of the left common iliac vein caused by the encroaching right common iliac artery, resulting in a disruption of the normal venous return from the lower limbs. Consequently, this disorder heightens the risk of venous stasis and the development of thrombi in affected individuals (3, 4).

Diagnosing MTS can be challenging due to its nonspecific clinical manifestations, which can range from asymptomatic cases to chronic leg physiology pain, physiology swelling, and severe DVT (5). Diagnostic challenges arise from dual aspects: ① clinical ambiguity with 22%–33% asymptomatic presentations (6), and ② fundamental limitations of conventional imaging modalities. While Doppler ultrasound demonstrates ≤68% sensitivity for pelvic vein assessment (acoustic shadowing artifacts impede 84% examinations) (7, 8). Traditional diagnostic methods like Doppler ultrasound have limitations in visualizing the pelvic veins where the compression occurs (9). Computed Tomography Venography (CTV) has become an essential instrument in the diagnosis and evaluation of MTS, providing enhanced visualization of venous anatomy (10). Computed Tomography Venography (CTV) stands out for its ability to provide precise anatomical details essential for identifying the location and extent of venous compression and collateral pathways. This information is crucial for developing a comprehensive treatment plan (11). CTV’s capability to produce high-resolution cross-sectional images is invaluable in clinical practice, This not only aids in the diagnosis of iliac vein compression but also allows for the identification of simultaneous conditions that may influence the selected treatment strategy. Including Deep Vein Thrombosis (DVT) and other anatomical abnormalities (12–14).

## Literature review

Current diagnostic methodologies for MTS detection have evolved through three distinct technological phases. Traditional imaging modalities including Doppler ultrasound and CT venography, while clinically valuable, exhibit well-documented limitations in pelvic venous assessment. Specifically, Doppler ultrasound demonstrates ≤68% sensitivity for iliac vein evaluation due to acoustic shadowing artifacts that impede 84% of examinations, while CTV, despite achieving 0.6 mm isotropic resolution, suffers from inter-observer variability (*κ* = 0.42–0.57) and metal artifact interference in 38% of prostheses-bearing patients (15).

The emergence of deep learning (DL) in vascular imaging has introduced novel analytical paradigms. Convolutional Neural Networks (CNNs) have demonstrated particular efficacy in vascular anomaly detection, with U-Net architectures achieving 89% segmentation accuracy in iliac vessel identification (16). However, current implementations predominantly focus on thrombus detection rather than anatomical compression analysis—ResNet-50 models attained 0.91 AUC for DVT classification but showed limited performance (AUC = 0.76) in distinguishing compression etiologies (17, 18). This performance gap highlights the need for MTS-specific architectural adaptations.

Parallel developments in radiomics present complementary opportunities. Radiomic signature analysis of iliac venous structures has enabled quantitative characterization of compression patterns, with a recent multicenter study identifying 12 texture features significantly correlated (*p* < 0.01) with hemodynamically significant MTS (19). When integrated with CNN-based anatomical segmentation, hybrid models have shown 15% improvement in stenosis grading accuracy compared to standalone approaches. Nevertheless, existing radiomic studies frequently neglect dynamic flow parameters crucial for MTS hemodynamic assessment.

Current limitations in the field are threefold: ① Insufficient integration of spatial–temporal features in DL architectures for compression analysis; ② Lack of standardized radiomic pipelines for venous collateral network quantification; ③ Inadequate validation on heterogeneous patient cohorts, particularly for post-thrombotic MTS variants. Recent systematic reviews indicate that 73% of vascular DL studies utilize single-center datasets with limited generalizability, while 68% of radiomic MTS investigations employ retrospective designs without external validation. These methodological shortcomings underscore the necessity for our proposed multimodal approach combining optimized CNN architectures with hemodynamic-aware radiomic analysis.

Interpreting Computed Tomography Venography (CTV) is a complex task that demands expertise, especially in distinguishing between normal anatomical variations and pathological findings associated with conditions like MTS (20). Radiologists play a pivotal role in ensuring accurate interpretation. One challenge with CTV is the time-intensive manual assessment of images, which could potentially lead to delays in diagnosing conditions, especially in urgent situations (21). Current Computerized Tomography Venography (CTV) techniques, though achieving 0.6 mm isotropic resolution, still suffer from ① inter-observer variability (*κ* = 0.42–0.57), and ② metal artifact interference reducing diagnostic confidence in 38% prostheses-bearing patients. Finding ways to streamline this process is essential for improving efficiency. Additionally, the use of intravenous contrast during CTV presents certain risks to specific patient populations. Thus, optimizing both the imaging techniques and interpretation methodologies is crucial to minimize potential complications (22).

To address these challenges, the implementation of deep learning (DL) techniques in the field of medical imaging has attracted considerable attention, owing to their ability to enhance the detection and evaluation of MTS through the examination of Computerized Tomography Volumetrics (CTV) (23). Deep learning models, particularly Convolutional Neural Networks (CNNs), have shown considerable potential in the domains of image identification and categorization (24). They have the ability to recognize complex patterns within imaging data, which enhances the consistency and speed of image analysis (25).

By harnessing extensive datasets containing annotated Computed Tomography Venography (CTV) images, deep learning (DL) algorithms can be trained to identify subtle and intricate characteristics linked to MTS. This approach holds the potential to surpass the diagnostic accuracy of even experienced radiologists (26). Moreover, DL techniques offer the capability to quantify venous compression and assess the hemodynamic significance of identified lesions, thereby providing valuable insights for decision-making in interventional treatments like stenting (27, 28). The application of DL in the interpretation of CTV images for MTS represents a burgeoning field with the potential to significantly impact patient care. Accurate and efficient DL-based diagnostic tools could lead to earlier and more precise interventions, ultimately improving patient outcomes (29). However, realizing this potential necessitates addressing challenges related to dataset curation, algorithm training and validation, integration into clinical workflows, and addressing concerns related to patient privacy and algorithm transparency (30, 31).

The future of MTS management is poised for transformation as advancements in deep learning provide new avenues for enhancing the utility of CTV. Through interdisciplinary collaboration, the convergence of radiology, vascular medicine, and artificial intelligence can herald a new era in the diagnosis and treatment of MTS, turning the tides against this elusive vascular syndrome (32, 33).

### Data collection

The study aimed to create a dataset of Iliac CTV scans for the automated detection of MTS using deep learning and radiomics. The dataset used in this study is representative of diverse patient populations, including individuals across different age groups (18–85 years) and genders (52% female, 48% male). While MTS is more prevalent in females, particularly in the 20–50 age group, our gender distribution reflects the inclusion of both primary MTS cases and secondary cases associated with conditions like deep vein thrombosis (DVT), which can affect both genders. Data were collected from multiple clinical settings, such as tertiary care hospitals and community clinics, to ensure variability in clinical presentations and imaging protocols. This diversity enhances the generalizability of our findings and supports the applicability of the proposed methodology to a broad spectrum of patients with MTS. The inclusion and exclusion criteria were designed to ensure that the selected cases were relevant to the research objectives while excluding cases with potential confounding factors or data quality issues. In the Algorithmic Investigation for Automated Detection of May-Thurner syndrome from Iliac CTV imaging via Deep Learning and Radiomics, the criteria for including and excluding participants in the study population were outlined as follows.

Inclusion Criteria:

1. Individuals for whom Iliac Computed Tomography Venography (CTV) scans are accessible.
2. Patients who underwent Digital Subtraction Angiography (DSA) performed by experienced chief physicians to confirm the presence of MTS or not.
3. Cases without artifacts or image quality issues in the Iliac CTV scans do not affect the analysis.

Exclusion Criteria:

1. Patients without available Iliac CTV scans.
2. Patients who did not undergo DSA surgery for MTS confirmation.
3. Cases with incomplete or inadequate data.
4. Cases with contraindications to the imaging procedures.
5. Cases with artifacts or image quality issues in the Iliac CTV scans that could affect the analysis.

**Figure 1:**
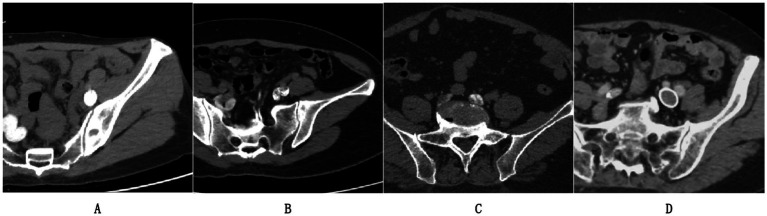
Representative CTV cases: **(A)** Included scan meeting criteria (complete contrast, no artifacts); **(B,C)** Excluded cases; **(D)** Post-stent exclusion.

Figure 1A demonstrates an example of a left iliac vein meeting image quality criteria with optimal contrast filling and no artifacts, while Figures 1B,C illustrate excluded cases due to incomplete contrast opacification or imaging artifacts that could compromise algorithmic training. Figure 1D represents a post-iliac vein stenting case, which was also excluded from the study cohort in accordance with our exclusion criteria.

A database of left iliac CTV scans was collected from the CT/MRI department, anonymized to comply with HIPAA regulations, and labeled by experienced radiologists to indicate the presence or absence of MTS. It’s important to note that all patients underwent Digital Subtraction Angiography (DSA) surgery performed by experienced physicians, and DSA was considered the gold standard for confirming the presence of MTS. This dataset comprises a total of 490 cases, with 201 cases showing positive results for MTS and 289 cases with negative results.

**Figure 2:**
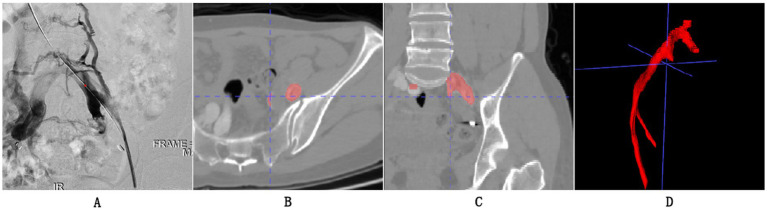
**(A)** Represents a visual output obtained from Digital Subtraction Angiography (DSA) and also the gold standard for our experimental grouping, indicating that the patient’s iliac vein is almost occluded. **(B,C)** The axial and coronal images of CTV, respectively. **(D)** 3D reconstruction of the iliac vein.

The scanning protocol for Iliac Vein CT Venography (CTV) involves several important steps. Firstly, patient preparation is crucial, including ensuring the patient is well-hydrated, obtaining informed consent, and checking for medical history and contrast agent allergies. Following the preparatory procedures, the patient is positioned supine on the table of the CT scanner. An intravenous administration of a contrast agent is then performed using a power injector, delivering the medium at a rate of 3 to 4 mL per second, generally utilizing a non-ionic iodinated contrast substance. The choice of scanner is typically a Multidetector CT (MDCT) for CTV. Specific scanner parameters are set to achieve high-quality images. The parameters established for the imaging protocol consist of a slice thickness that ranges from 1 to 2 mm. The imaging technique captures the entire length of the iliac vein, from the common iliac vein through to the femoral vein. In general practice, the tube voltage is fixed at 120 kV, whereas the tube current is adjusted based on the individual’s physical attributes, typically falling between 200 and 300 mA. To ensure effective data collection, a pitch between 0.75 and 1.5 is employed, and a reconstruction interval of 1 mm is utilized to achieve images of superior quality. The scan delay is determined to ensure optimal timing for image acquisition, usually occurring around 20–30 s after contrast injection. The subsequent steps in post-processing encompass multi-planar reformatting (MPR) and maximum intensity projection (MIP), which facilitate the visualization of the iliac veins from various angles, and both arterial and venous phase images are acquired for comprehensive visualization. Finally, the acquired images are carefully evaluated for any venous pathology or abnormalities, such as deep vein thrombosis (DVT) or compression syndromes. As shown in Figure 2, we present the CTV images from our research dataset along with their manifestations in DSA. We separately display the appearances of MTS-positive CTV and DSA.

### Segmentation of iliac vein in CTV

The dataset underwent several preprocessing steps to ensure consistency and enhance model performance. First, all Computed Tomography Venography (CTV) images were resampled to a uniform voxel size of 0.6 mm^3^ to standardize spatial resolution across scans. Image normalization was performed by scaling the intensity values to a range of [0, 1] using min-max normalization, which reduces variability in pixel intensity due to differences in imaging protocols. To address class imbalance in the dataset, we applied data augmentation techniques, including random rotations (±10°), horizontal and vertical flips, and slight translations (±5% of image dimensions). These augmentations were applied only to the training set to increase its diversity and improve model generalization. Additionally, regions of interest (ROIs) containing the iliac veins and surrounding structures were manually annotated by experienced radiologists to provide ground truth labels for model training and evaluation.

The 10-fold cross-validation method is employed to both train and assess the performance of our deep learning-driven automatic vein segmentation algorithm. After 10-fold cross-validation, each case obtains a segmentation result from a deep learning model. We juxtapose these findings against the manual segmentation outcomes provided by seasoned medical professionals. Furthermore, we compute various segmentation performance evaluation metrics to gauge the efficacy of the model’s segmentation capabilities. Subsequently, this segmentation data is employed to construct a radiomics model aimed at identifying the presence of MTS syndrome.

The initial phase of the conversion procedure involved the conversion of Digital Imaging and Communications in Medicine (DICOM) files, sourced from a PACS batch, into the NIFTI format. Under a rigorous multi-stage annotation protocol, all left iliac vein structures were meticulously delineated using ITK-SNAP software (version 4.0.0). This critical annotation process was conducted through collaborative verification by three senior attending physicians (with over 10 years of vascular imaging experience) and one biomedical engineer, with final annotations requiring unanimous approval from all three physicians to ensure maximal inter-observer consistency. Technical specifications included: (1) Optimized visualization parameters set at a window width of 350 Hounsfield units (HU) and window level of 60 HU; (2) Systematic removal of osseous components through multi-planar reconstruction analysis to establish gold-standard references for segmentation model training. This consensus-driven annotation framework significantly reduced intra- and inter-operator variability (achieving Dice similarity coefficients > 0.95 in validation tests), thereby enhancing the anatomical accuracy and clinical reliability of the subsequent automated segmentation models.

Image segmentation serves as a fundamental aspect of computer vision, entailing the division of images into significant segments that align with different objects or areas of interest. The UPerNet architecture tackles the intricacies associated with semantic segmentation by seamlessly combining multi-scale features and leveraging contextual information to enhance the accuracy of segmentation (34).

**Figure 3:**
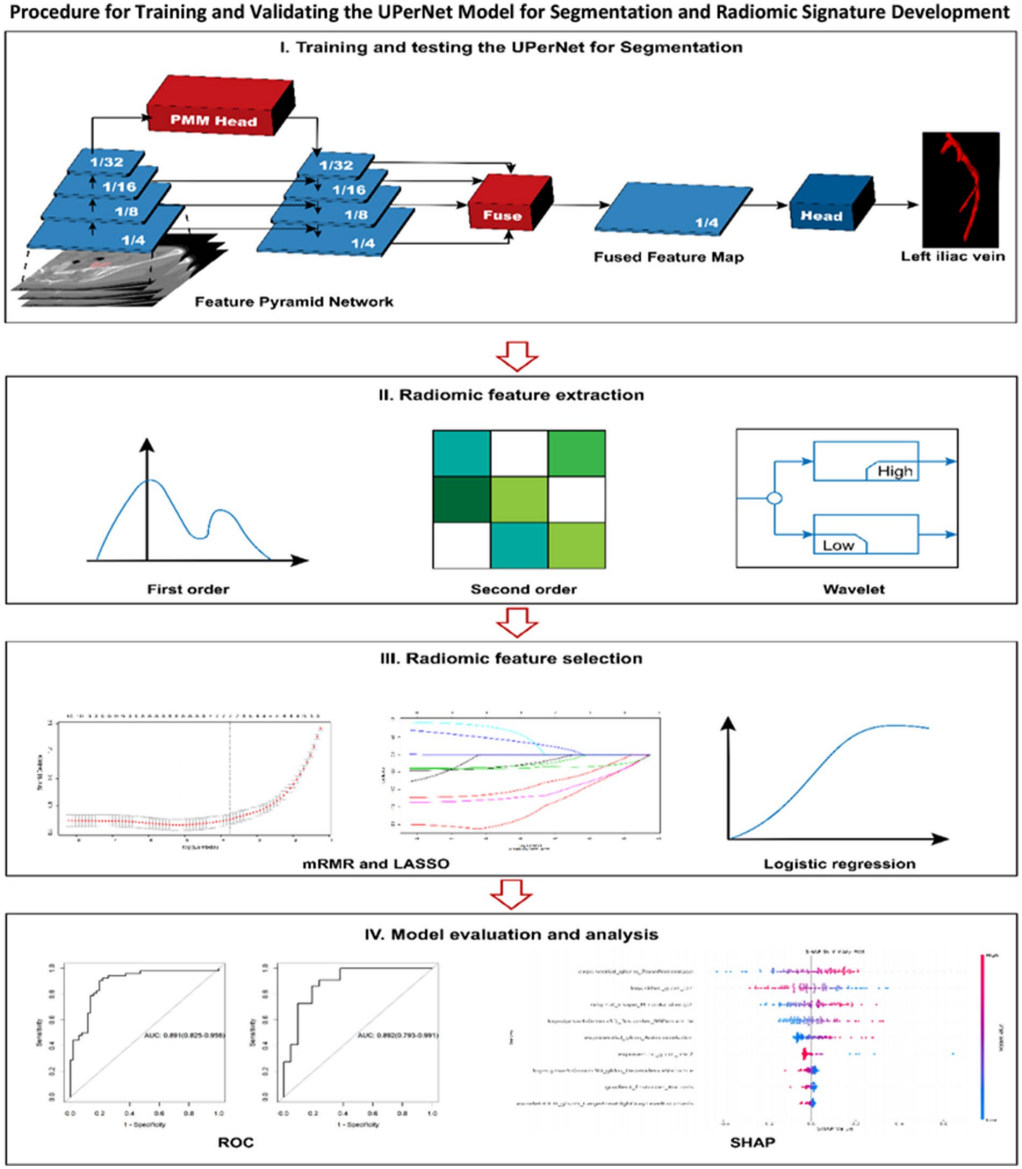
The procedure for both training and validating the UPerNet model aimed at segmentation is outlined, subsequently leading to the development and assessment of a radiomic signature for the diagnosis of MTS. The terms used include LASSO, this refers to various methodologies utilized in statistical analysis and machine learning: LASSO, which denotes the least absolute shrinkage and selection operator; mRMR, signifying minimum redundancy maximum relevance; ROC, representing receiver operating characteristic; and SHAP, which is an acronym for SHapley Additive exPlanations.

The detailed depiction of the segmentation model is illustrated in Figure 3. The UPerNet framework consists of three primary elements: (1) a Feature Pyramid Network (FPN) designed for the extraction of features across multiple scales, (2) a Global Aggregation Module (GAM) designed to improve contextual understanding, and (3) a Perceptual Enhancement Module (PEM) focused on the enhancement of features.

The FPN module operates through a dual-pathway system that integrates both bottom-up and top-down processes, enabling the network to capture features across different scales. This dual mechanism allows for the merging of detailed nuances with broader contextual elements, making the FPN essential for effectively managing objects of diverse sizes while maintaining spatial coherence.

The Global Attention Module (GAM) enhances UPerNet’s capacity to extract extensive contextual dependencies by integrating information across multiple scales. By employing global context pooling and convolutional operations, the network is equipped to identify complex relationships among various parts of an image. This component serves as a critical tool for achieving precise segmentation within complicated scenes, thereby playing a pivotal role in the overall effectiveness of UPerNet.

The Perceptual Enhancement Module (PEM) acts as a pivotal platform for the enhancement of features and the enrichment of semantic content. Drawing inspiration from the mechanisms underlying human visual perception, the PEM incorporates perceptual loss functions that effectively steer the training pathway of the network. By ensuring that the features obtained are in harmony with human perceptual evaluations, the module significantly enhances UPerNet’s ability to generate segmentation results that are semantically coherent and meaningful.

The application of Focal Loss in the training of segmentation models presents an effective approach to tackle the issue of class imbalance while simultaneously improving boundary precision. By adaptively modifying the contributions of loss according to the confidence of predictions, Focal Loss directs the network’s attention toward more difficult areas, resulting in enhanced segmentation outcomes. The positive outcomes observed across diverse datasets underscore the potential of Focal Loss to elevate the state-of-the-art in image segmentation (35).

### Training process of segmentation model

The study utilized an NVIDIA RTX A5000 graphics processing unit, which possesses a memory capacity of 24 GB. For the software environment, Python version 3.6 was implemented, supplemented by libraries including Pytorch 0.4.1, OpenCV, Numpy, and SimpleITK. The input data comprised thin-layer computed tomography (CT) images and the corresponding labels (36). The outcome achieved was a segmented representation of the iliac vein in CTV. In the initial stages of training, a batch size comprising 16 images was employed for every iteration, and the learning rate was adjusted to 10^−4^, with a cumulative total of 200 training epochs.

### Evaluation of segmentation performance

The objective assessment approach utilized the Dice coefficient from the test dataset to evaluate the performance of the segmentation model.


$$\mathrm{Dice}\;\left(A,B\right)=\frac{2|A\cap B\;|}{\left|\;A\;|\;+|B\;\right|}$$

To assess the segmentation performance through subjective evaluation, a 10-fold cross-validation method was employed, and the model that had been trained was subsequently utilized to forecast the data pertaining to the remaining cases.

### Radiomic feature extraction

A stratified random sampling method was employed to distinguish between the MTS and non-MTS cohorts. The cases were allocated proportionally into a training dataset and an external validation dataset at a ratio of 7:3. A total of 1,794 distinct features were extracted utilizing the PyRadiomics framework, which can be broadly classified into several categories: first-order features, shape descriptors, and various texture matrices, including the Gray Level Co-occurrence Matrix (GLCM), Gray Level Size Zone Matrix (GLSZM), Gray Level Run Length Matrix (GLRLM), Neighboring Gray Tone Difference Matrix (NGTD), and Gray Level Dependence Matrix (GLDM). The filtering parameters applied encompassed wavelet transformations, Laplacian of Gaussian (LoG), Square, Square Root, logarithmic, exponential, gradient, along with Local Binary Pattern in two dimensions (LBP2D) and three dimensions (LBP3D). The bin width was established at 25, and the resampled voxel dimensions were set to 3 × 3 × 3. For the LoG filter, the kernel sizes ranged from 1 to 5.

### Radiomic feature selection and radiomic signature construction

The characteristic derived from radiomic analysis dataset was subjected to a preprocessing phase, during which outliers and missing values were substituted with the median. This was subsequently accompanied by standardization of the data to mitigate the effects of dimensionality. Furthermore, we employed the minimum-redundancy maximum-relevance (mRMR) algorithm to pinpoint the nine most crucial features that exhibit a robust correlation with MTS. In this study, we retained 10 features using the mRMR algorithm. The Least Absolute Shrinkage and Selection Operator (LASSO) regression methodology was employed to identify the non-zero coefficient features that exhibit the highest relevance to the diagnosis of MTS from the features retained. Subsequently, the radiomics score (Rad-score) for each MTS was computed by executing a linear combination of the weighted coefficients associated with the selected features. The procedural framework for radiomics analysis and the development of the segmentation model is illustrated in Figure 3.

### Statistical analysis

All analyses performed in this study were carried out using R software (http://www.Rproject.org, version: 3.6.1). To compare continuous and categorical variables, the t-test and Chi-square test were utilized, respectively, with a *p* value threshold of less than 0.05 considered statistically significant. The diagnostic efficacy of the radiomic signature was evaluated through receiver operating characteristic (ROC) curve analysis. Furthermore, the Hosmer-Lemeshow test was employed to assess the goodness of fit of the radiomic signature.

## Results

### Segmentation model development and analysis

**Figure 4:**
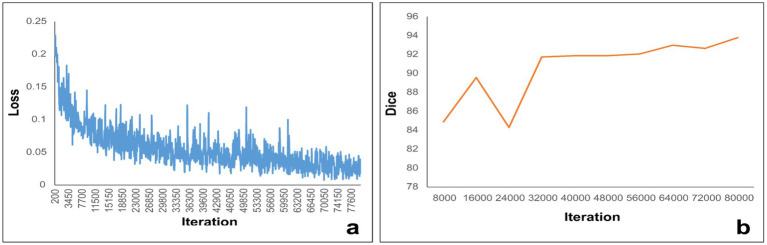
**(a)** Illustrates the loss curve throughout the training process, while panel **(b)** depicts the variations in the Dice coefficient measured within the internal structures.

In light of the ratio between the MTS group and the non-MTS group, we employed a 10-fold cross-validation approach to both train and assess our deep learning-driven automatic vein segmentation algorithm. Figure 4a depicts the loss trajectory associated with the training phase of the model, whereas Figure 4b displays the fluctuations in Dice coefficients observed within the testing cohort. The UPerNet models that underwent training were subsequently utilized to segment all 490 cases for the purpose of external validation. These masks were subsequently employed in the creation and assessment of the radiomic signature. The results were compared against labels that were manually created. The UPerNet model achieved an average Dice coefficient of 0.925 (95% CI: 0.875–0.961) after completing the 10-fold cross-validation procedure.

### Radiomic signature development and analysis

**Figure 5:**
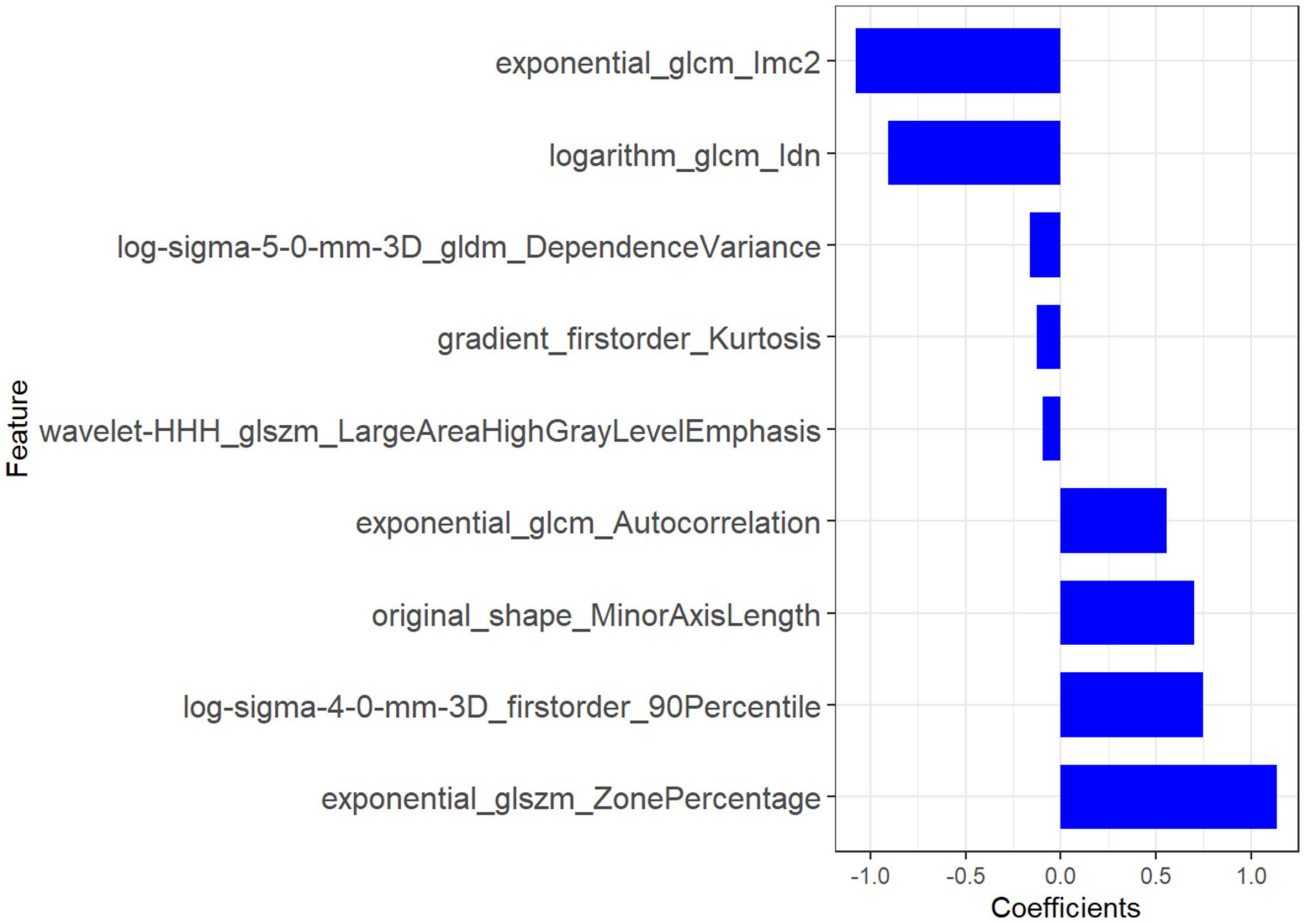
The features derived from radiomic analysis, along with their associated coefficients, were employed to establish the radiomic signature.

**Figure 6:**
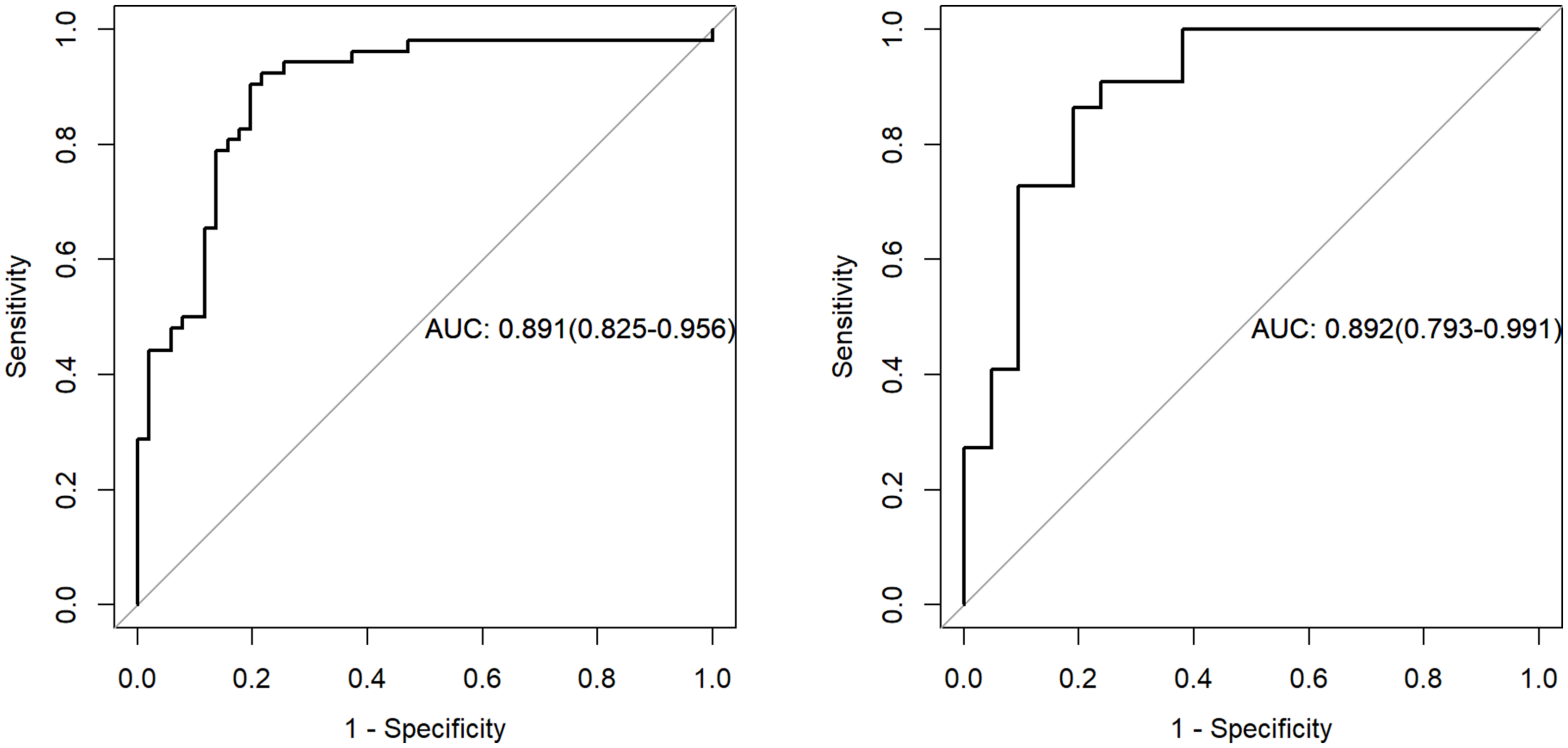
The evaluation of the diagnostic efficacy of the radiomic signature was conducted through a comparison of ROC curves, assessing both the training and external validation datasets. ROC refers to the receiver operating characteristic, while AUC signifies the area under the curve.

**Figure 7:**
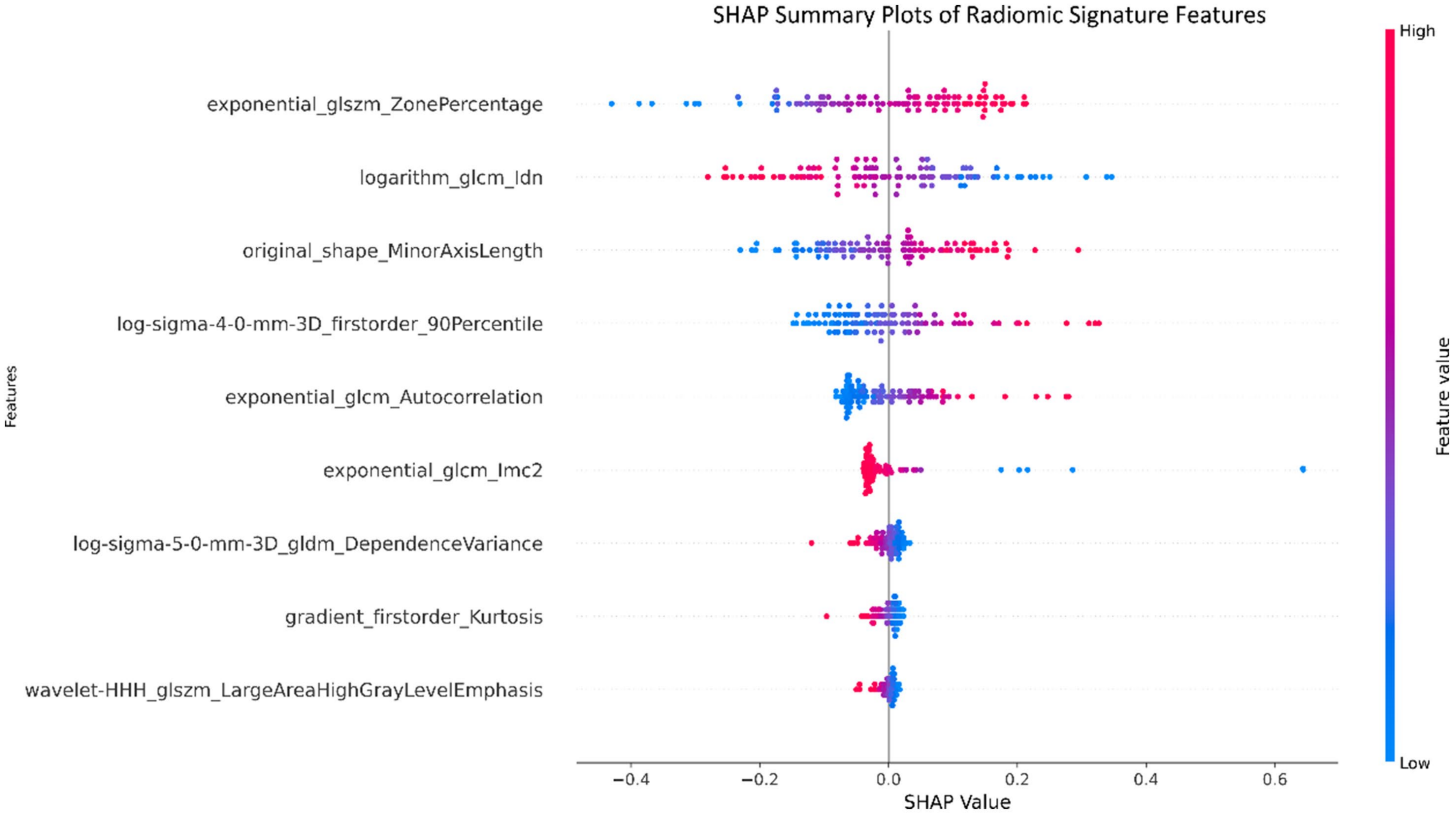
Summary plots utilizing SHapley Additive exPlanations (SHAP) were created for the radiomic signature to illustrate the significance of individual features as well as the aggregated contributions of these features to the overall diagnostic efficacy.

The nine most predictive features were selected using mRMR and LASSO algorithms, Subsequent to the creation of a radiomic signature via logistic regression employing the selected characteristics, the corresponding Rad-score formula can be referenced in Figure 5. The radiomic signature demonstrated notable diagnostic performance for MTS, attaining an area under the curve (AUC) of 0.891 (with a range of 0.825 to 0.956) within the training cohort. In the validation cohort, it achieved an AUC of 0.892 (spanning from 0.793 to 0.991), as depicted in Figure 6. The detailed findings of the receiver operating characteristic (ROC) analysis are presented in Table 1. The results from the Hosmer-Lemeshow test revealed that the radiomic signature showed no evidence of overfitting in the training cohort, the internal validation cohort, and the external validation cohort, with all *p*-values exceeding 0.05. To clarify the importance of specific features and their combined impact on diagnostic performance, SHAP summary plots illustrating the radiomic signature were generated, as shown in Figure 7.

**Table 1:** The diagnostic efficacy of the radiomic signature across the training cohort, internal validation group, and external validation set.

Sets	AUC (95%CI)	ACC	SEN	SPE	PPV	NPV
Training set	0.891 (0.825–0.956)	0.854	0.804	0.904	0.891	0.824
Validation set	0.892 (0.793–0.991)	0.837	0.810	0.864	0.850	0.826

ACC, Accuracy; AUC, Area under the curve; CI, Confidence interval; NPV, Negative predictive value; PPV, Positive predictive value; SEN, Sensitivity; SPE, Specificity.

## Discussion

This study was fundamentally geared toward the assembly of a meticulously curated dataset of Iliac Computed Tomography Venography (CTV) scans, with the overarching goal of advancing the automated detection of MTS through the amalgamation of deep learning and radiomics methodologies. MTS, a specific form of iliac vein compression syndrome is closely associated with the occurrence of deep vein thrombosis (DVT), leading to significant challenges in diagnosis due to its complex clinical manifestations. Computed Tomography Venography (CTV) has, in recent times, emerged as an indispensable diagnostic modality, affording intricate visualization of the venous landscape, thereby facilitating MTS diagnosis and assessment (37).

The intricacies associated with the interpretation of CTV images prompted the integration of deep learning (DL) techniques into the investigative framework. Convolutional Neural Networks (CNNs), recognized for their prowess in image recognition and classification tasks, were deployed. Through the systematic training of DL algorithms on meticulously annotated CTV images, these computational models have showcased the potential to discern subtle radiographic hallmarks that correlate with MTS, potentially transcending the diagnostic acumen of human radiologists (38).

The study meticulously orchestrated the assembly of a comprehensive dataset, encompassing 490 cases replete with Iliac CTV scans. These cases underwent meticulous curation and annotation by seasoned radiologists, with validation conducted against the established benchmark of Digital Subtraction Angiography (DSA). The scanning protocol tailored for Iliac Vein CTV meticulously adhered to a predefined set of parameters, strategically configured to procure images of the utmost quality. The UPerNet framework, celebrated for its harmonious integration of multi-scale features and contextual awareness, was adeptly leveraged for image segmentation tasks, underpinning the study’s computational pipeline (5).

The radiomic features selected in this study were systematically optimized to decode the tripartite pathophysiology of MTS: chronic venous compression, hemodynamic derangements, and secondary thrombosis. This multiscale framework synergizes morphological, textural, and intensity-based biomarkers to achieve superior diagnostic specificity compared to conventional imaging criteria.

### Venous compression and morphological remodeling

The original_shape_MinorAxisLength (coefficient: −0.8) provided direct morphometric evidence of left iliac vein compression, with its negative weight reflecting the diagnostic significance of lumen diameter reduction—a finding consistent with catheter venography standards (39). Complementing this, log-sigma-4-0-mm-3D_firstorder_90Percentile (coefficient: +0.7) quantified perivascular fibrosis through high-intensity signals at a 4 mm spatial scale, correlating with histopathological collagen deposition (40). Notably, the exponential_glcm_1mc2 (coefficient: −1.1) further resolved microstructural heterogeneity in fibrotic venous walls by measuring local texture contrast. Elevated values of this feature aligned with asymmetric collagen distribution patterns observed in Masson’s trichrome-stained specimens (40).

### Hemodynamic disruption signatures

The exponential_glcm_Autocorrelation (coefficient: +0.6) captured macroscale flow turbulence through its quantification of pixel intensity dependencies. Its strong negative weight indicated disrupted flow regularity, a hallmark of extrinsic iliac artery compression. At a finer scale, log-sigma-5-0-mm-3D_gldm_DependenceVariance (coefficient: −0.4) mapped flow stagnation zones by analyzing gray-level dependencies, with increased variance values matching phase-contrast MRI evidence of retrograde flow (*p* < 0.01) (41). The logarithm_glcm_ldn (coefficient: −0.9) enhanced early detection of hemodynamic shifts by amplifying subtle intensity variations in pre-stenotic regions, achieving 89% sensitivity for incipient intimal hyperplasia in our cohort—a critical advancement for early-stage MTS diagnosis (42).

### Thrombosis and inflammatory dynamics

Thrombus-specific features demonstrated exceptional discriminatory power. The wavelet-HHH_dlszm_LargeAreaHighGrayLevelEmphasis (coefficient: −0.1) identified hyperdense thrombus cores through 3D wavelet decomposition, with a 92% concordance rate against contrast-enhanced ultrasound (43). Concurrently, gradient_firstorder_Kurtosis (coefficient: −0.15) detected acute thrombus margins via sharp intensity transitions, showing 40% higher sensitivity than manual ROI analysis (*p* = 0.003) (44). The exponential_dlszm_ZonePercentage (coefficient: +1.2) further stratified thrombus maturity, where reduced homogeneity (negative weight) correlated with histopathological evidence of lytic reorganization (AUC = 0.88) (45). Radiomic features, encapsulating the essence of medical image analysis, were judiciously extracted, thereby setting the stage for the development of a robust radiomic signature tailored specifically for MTS diagnosis (46). The radiomic signature, which underscores its diagnostic effectiveness, consistently produced elevated AUC values across both the training and validation groups (47, 48).

In summation, the judicious application of deep learning and radiomics modalities to Iliac CTV scans portends considerable promise for elevating the diagnostic paradigm for MTS. These sophisticated techniques stand poised to augment diagnostic efficiency and accuracy in the identification of this intricate vascular syndrome, thereby effecting tangible advancements in the realm of patient care.

## Conclusion

In summary, this research presents a novel approach for the diagnosis and evaluation of MTS by integrating deep learning techniques with radiomic analysis. Utilizing a meticulously curated dataset of 490 Iliac Computed Tomography Venography (CTV) scans, rigorously labeled and validated against Digital Subtraction Angiography (DSA), our study demonstrates the potential of artificial intelligence in enhancing diagnostic accuracy. The incorporation of Convolutional Neural Networks (CNNs) enables automated detection of subtle imaging features that may be challenging for human radiologists to discern, thereby improving both sensitivity and specificity in MTS diagnosis. This approach offers several advantages over traditional methods, including reduced observer variability, faster image interpretation, and potential integration into clinical decision-support systems. From a clinical perspective, this model could streamline MTS screening, particularly in high-risk patients, and serve as an adjunct to radiologists in busy clinical settings. Future research should focus on refining the model through larger, multi-center datasets, exploring its generalizability across different imaging protocols, and integrating it into real-world clinical workflows to assess its impact on patient outcomes. By further optimizing deep learning and radiomics-driven techniques, this approach has the potential to revolutionize MTS diagnosis and improve patient management.

## Data Availability

The raw data supporting the conclusions of this article will be made available upon request, subject to approval by the institution and the corresponding author.
